# Clinical Efficacy of Endoscopic-Assisted Resection of Single-Segment Ossification of the Posterior Longitudinal Ligament in the Treatment of Thoracic Spinal Stenosis

**DOI:** 10.3389/fsurg.2022.897182

**Published:** 2022-05-06

**Authors:** Xingchen Li, Honghan Huang, Zhong Zheng, Yunxuan Liu, Guicai Wei, Xiaoxin Chen, Yusheng Xu

**Affiliations:** ^1^ Department of Orthopedics Surgery, The First Affiliated Hospital of Zhengzhou University, Zhengzhou, China; ^2^ Department of Spine Surgery, Fuzhou Second Hospital Affiliated to Xiamen University, Fuzhou China

**Keywords:** minimally invasive, spinal endoscopy, posterior longitudinal ligament ossification, thoracic spinal stenosis, TSS, OPLL (ossification of the posterior longitudinal ligament)

## Abstract

**Objective:**

To explore the clinical efficacy, characteristics and safety of endoscopic-assisted resection of single-segment posterior longitudinal ligament ossification in the treatment of thoracic spinal stenosis (TSS).

**Method:**

Fifteen TSS patients, including 6 males and 9 females aged 43–70 years treated with endoscopic-assisted resection of single-segment posterior longitudinal ligament ossification through the transfacet joint approach by our team from November 2016 to June 2020 were retrospectively analyzed. The operation time, intraoperative blood loss, and postoperative complications were recorded. The VAS score, ODI and JOA score (full score, 11 points) were recorded before the operation, after the operation and at the last follow-up to evaluate the clinical efficacy and calculate the improvement rate.

**Results:**

The ventral side of the spinal cord was decompressed in all patients, providing improvements in neurological symptoms and significant pain relief. The mean follow-up time was 20.27 ± 3.87 months. Mean operation time, intraoperative blood loss, and hospitalization time were found to be 84.80 ± 13.23 min, 36.33 ± 7.41 mL, 5.13 ± 1.02 days; respectively.The JOA score at the last follow-up was 8.6 ± 1.25, which was significantly better than the preoperative (5.53 ± 1.20) and postoperative (6.87 ± 1.31) scores (*p* < 0.05). The mean JOA score improvement rate was 56.5 ± 18.00%. The JOA score improvement rate classification at the last follow-up was excellent in 3 cases, good in 8 cases, effective in 3 cases, and no change in 1 case; for an effective rate of 93.33%. The VAS score significantly decreased from 6.67 ± 1.01 preoperatively to 3.47 ± 0.88 postoperatively and 1.73 ± 0.67 at the last follow-up (*p* < 0.05). The ODI significantly decreased from 72.07 ± 6.08 preoperatively to 45.93 ± 5.01 postoperatively and 12.53 ± 2.33 at the last follow-up (*p* < 0.05). Dural rupture occurred in 2 patients during the operation; 1 patient experienced neck discomfort during the operation, which was considered to be caused by high fluid pressure and was relieved by massage and by lowering the height of the irrigation fluid. No cases of cerebrospinal fluid leakage, wound infection or other complications occurred.

**Conclusion:**

Endoscopic-assisted resection of posterior longitudinal ligament ossification through the facet joint approach is a safe and effective method for the treatment of TSS.

## Introduction

Thoracic spinal stenosis (TSS) is a degenerative disease that progresses slowly and can remain asymptomatic for a long time, but the delay in diagnosis results in irreversible damage to the nervous system at the time of diagnosis (1–4). The pain caused by TSS also seriously affects the work and life of patients. Therefore, the treatment of TSS is extremely important. Although there is no unified principle or standard for the treatment of TSS, relevant scholars generally believe that the clinical effect of conservative treatment for TSS is poor. As a result, patients with TSS should undergo surgery soon after diagnosis (5).

In patients with TSS, ossification of the posterior longitudinal ligament (OPLL) is the main cause of ventral spinal cord compression (6–8). A large number of scholars have reported that the traditional open surgical treatment method has disadvantages, including the severe trauma, many potential complications, and unstable efficacy (9), and this surgical method is becoming increasingly unsuitable for many patients. With the development of minimally invasive techniques, endoscopic spinal surgery has been widely used in the treatment of diseases affecting the cervical and lumbar spine, with good clinical results (10, 11). At the same time, some scholars have applied endoscopic techniques in the treatment of OPLL of the thoracic spine and have also achieved good surgical results (12). Therefore, endoscopic-assisted resection of OPLL in the thoracic spine for the treatment of TSS has gradually become a research focus.

However, there have been few reports on the treatment of TSS by an endoscopic-assisted transfacet joint approach. We used an endoscopic-assisted transfacet joint approach for the resection of OPLL and discussed the characteristics, clinical efficacy and safety of this operation in the treatment of TSS.

## Materials and Methods

### Patients

Fifteen patients with TSS treated by endoscopic-assisted resection of single-segment posterior longitudinal ligament ossification through the transfacet joint approach by our team from November 2016 to June 2020 were retrospectively analyzed. Common clinical manifestations were chest and back pain, girdle sensation, numbness in the saddle area, bowel and bladder dysfunction, numbness and weakness of both lower extremities, unsteady walking, sensation of stepping on cotton while walking, increased muscle tone, and tendon hyperreflexia.

### Inclusion and Exclusion Criteria

The inclusion criteria were as follows: 1. diagnosis of single-segment TSS caused by OPLL made according to the patient’s medical history, clinical manifestations and imaging findings; 2. dysfunction at and below the affected segment, with a serious impact on quality of life; 3. persistence or progression of symptoms during conservative treatment at regular institutions; and 4. complete follow-up data.

The exclusion criteria were as follows: 1. TSS caused by a tumor, infection, fracture or deformity of the spine; 2. history of thoracic spine surgery or thoracic spine instability; 3. paraplegia or other underlying disease precluding surgery; 4. TSS of two or more segments or cervical or lumbar spine disease; or 5. TSS was caused by intervertebral disc herniation and ossification of the ligamentum flavum.

### Preprocedural Imaging

Several imaging examinations were performed before the operation. 1. Frontal and lateral X-rays of the thoracic spine and double oblique X-rays were obtained, if necessary. 2. Thin-slice computed tomography (CT) of thoracic vertebral lesions was performed to determine the position, extent, and severity of compression, as well as to identify the presence of dural sac adhesion or ossification to prepare for dural sac rupture and cerebrospinal fluid leakage in advance. 3. Magnetic resonance imaging (MRI) of the thoracic spine was performed to examine the compression site for a high signal intensity on T2-weighted imaging (T2WI) (13, 14) and to predict the clinical efficacy and prognosis. Based on the clinical manifestations combined with the preoperative imaging results, the surgical approach was designed. The thickness, length and bypass distance of the compression site were measured in advance, and an individualized surgical plan was formulated for each patient.

### Choice of Surgical Approach

The surgical approach was determined preoperatively based on the CT and MRI findings of each patient. Patients with OPLL that deviated to one side and did not extend beyond the midline underwent surgery with a unilateral approach for decompression. Patients who had OPLL that was central, wide-based and extend beyond the midline underwent surgery with a bilateral approach for decompression.

### Surgical Procedure

**Figure 1:**
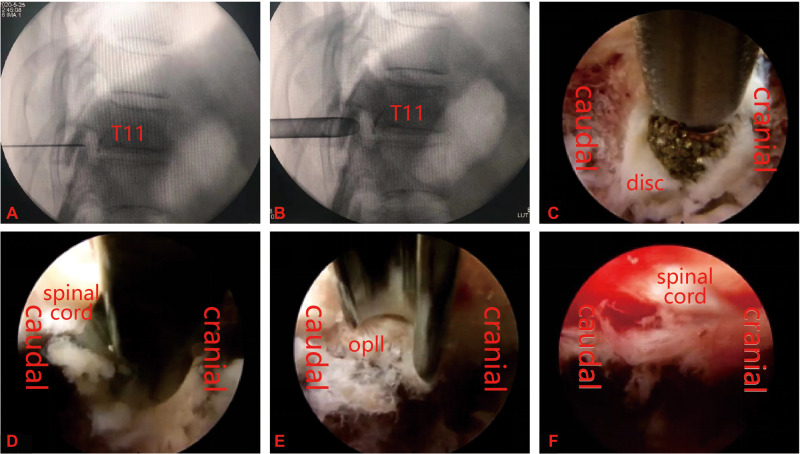
(**A**,**B**) Percutaneous puncture to the dorsal side of the facet joint, followed by insertion of a working cannula. (**C**–**E**) Removal of the ossified posterior longitudinal ligament by decompression using a power drill system, blue forceps and osteotome. (**F**) Complete removal of ossified tissue and full decompression of the meninges after the operation.

**Figure 2:**
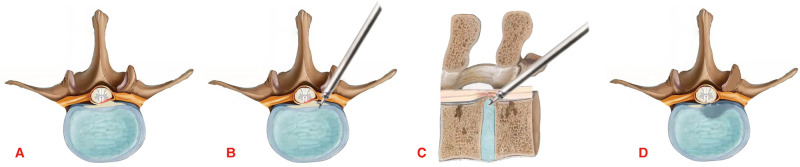
Diagram of the surgical procedure. (**A**) The spinal cord is compressed by the ossified posterior longitudinal ligament. (**B**,**C**) Removal of the ossified posterior longitudinal ligament through the facet joint approach. (**D**) The ossified posterior longitudinal ligament was completely removed and the ventral side of the spinal cord was completely decompressed.

The patient was placed such that the lesion was facing upward, with pillows placed under the waist and armpits. Oxygen was inhaled, and electrocardiography, blood pressure, and blood oxygen saturation monitoring were performed. To relieve pain and keep the patient awake, the anesthesiologist administered dexmedetomidine (0.2–0.7 µg/kg/min) and sufentanil (0.1 μg/kg). C-arm X-ray was used to locate the surgical segment. After accurate positioning, the needle insertion point was marked on the body’s surface, usually 5–8 cm lateral to the midline of the spinous process. Lidocaine was infiltrated layer by layer to establish anesthesia to the posterior aspect of the facet joint. Under fluoroscopic guidance, an 18-gauge puncture needle was inserted. After the guide wire was inserted, the puncture needle was removed, and a skin incision of approximately 1 cm with the needle insertion point as the midpoint was created. In turn, the dilation catheter was inserted along the guide wire, which was placed close to the lateral edge of the lamina or the bone at the facet joint, the endoscopic working channel was inserted along the dilation catheter, and the dilation catheter was pulled out. Fluoroscopy was performed again to ensure correct positioning of the channel. The guide wire was observed to be at an angle of 50° to 70° with respect to the sagittal plane of the spine. The endoscope was then inserted through the working channel, and blunt dissection of the soft tissue at the facet joint was performed. The tissue was separated along the lateral edge of the facet joint, the facet joint and the upper edge of the pedicle of the lower vertebral body were separated, and an external serrated trephine was placed on the selected facet joint. After removing the lateral edge of the tip of the inferior articular process, the exposed articular cartilage could be observed; the cartilage was circumscribed, the dorsal side of the superior articular process was exposed, the superior articular process was trephined, and the dorsal lateral edge of the ligamentum flavum or the pedicle was exposed. Then, the dura mater and the intercostal nerve root were separated from the ventral side, using an inclined working cannula to protect the nerve root, and the intervertebral disc and ossified posterior longitudinal ligament were exposed. Biopsy forceps were used to cut the annulus fibrosus. Then, the connection between the edge of the ossified posterior longitudinal ligament and the posterior edge of the vertebral body was severed, and the ossified posterior longitudinal ligament was polished from the ventral side of the posterior longitudinal ligament to a thin and translucent state with a diamond drill; a nerve dissector was placed in the space between the ossified posterior longitudinal ligament and the dura and used to press toward the ventral side until the ossified posterior longitudinal ligament was completely removed (Figure 1). After the compressive material was removed, the dural sac was inflated, good pulsation was confirmed, and the nerve root was fully released. Postoperative radiofrequency coagulation was used to stop bleeding. The working cannula was removed, and the incision was sutured (Figure 2).

### Postoperative Management

No drain was placed after the operation. The pillows were removed for 8 h, and the patient was placed in a supine position. The vital signs and sensory and motor conditions of the limbs were closely observed. Patients were encouraged to ambulate the day after surgery; wear thoracic and lumbar supports for 3 months after surgery to facilitate wound repair; and avoid prolonged sitting, bent-over weight-bearing, twisting and other strenuous activities. Antibiotics were routinely administered for 3 days. CT and MRI reexaminations were performed 3 days after the operation.

### Clinical Evaluation

The visual analog scale (VAS) pain score, Oswestry Disability Index (ODI) and modified Japanese Orthopedic Association (JOA) score (full score, 11 points) (Table 1) were used to evaluate the patients preoperatively, 3 days after the operation and at the final follow-up. After the evaluation was performed, the JOA score improvement rate was calculated using the following formula: RR = (postoperative score - preoperative score)/(11 - preoperative score) × 100%. The JOA score improvement rate was then classified as excellent (75%–100%), good (50%–74%), effective (25%–49%), and no change or deterioration (0%–24%) (15). Perioperative data, including the operation time, intraoperative blood loss and postoperative complications, were recorded.

**Table 1:** Summary of the JOA scoring system for the assessment of thoracic myelopathy.

Neurological status	Score
Lower-limb motor dysfunction	
No dysfunction	4
Lack of stability and smooth reciprocation of gait	3
Able to walk on flat floor with walking aid	2
Able to walk up/downstairs with handrail	1
Unable to walk	0
Lower-limb sensory deficit	
No deficit	2
Mild sensory deficit	1
Severe sensory loss or pain	0
Trunk sensory deficit	
No deficit	2
Mild sensory deficit	1
Severe sensory loss or pain	0
Sphincter dysfunction	
No dysfunction	3
Minor difficulty in micturition	2
Marked difficulty in micturition	1
Unable to void	0

### Statistical Analysis

Statistical analysis was performed using SPSS 21.0. Statistical data conforming to a normal distribution are expressed as $\bar{x}\pm s$. The preoperative and postoperative ODIs were analyzed by paired-samples *t*-test, and the VAS and JOA scores were analyzed by rank-sum test. *p* < 0.05 was considered statistically significant.

## Results

**Figure 3:**
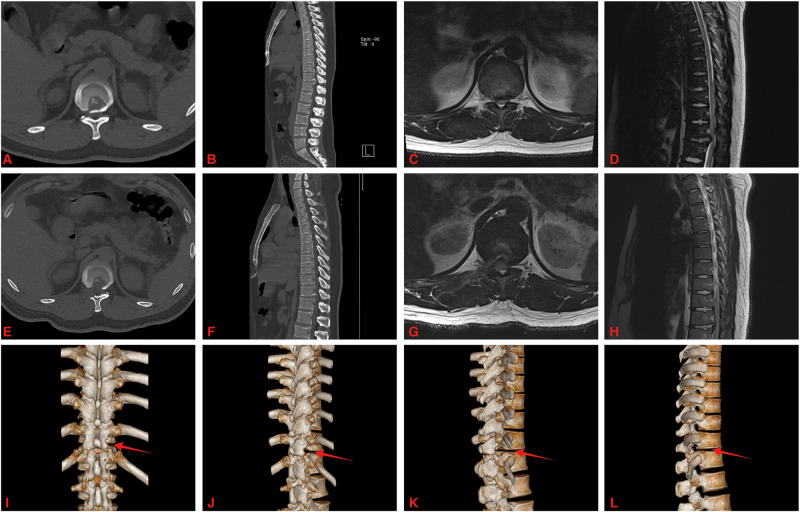
A 46-year-old male with OPLL at T11-12. (**A**–**D**) Preoperative CT and MRI showed T11-12 posterior longitudinal ligament ossification and spinal cord was compressed. (**E**–**H**) Postoperative CT and MRI showed that the ossified posterior longitudinal ligament was removed and the ventral side of the spinal cord was completely decompressed. (**I**–**L**) The three-dimensional reconstruction of the CT images showed right side facet joint of T11-12 was resected (red arrow).

All operations were successfully completed. Among the 15 patients, there were 6 males and 9 females; the age ranged from 43 to 70 years, with an average of 56.7 ± 8.4 years. The follow-up time ranged from 13–27 months, with an average of 20.27 ± 3.87 months. The ventral spinal cord was decompressed in all patients with the aid of spinal endoscopy. Postoperative imaging showed full spinal decompression and ossified posterior longitudinal ligament removal in all patients. Twelve patients underwent surgery with a unilateral approach, and 3 patients underwent surgery with a bilateral approach (Figure 3).

The mean operation time was 84.80 ± 13.23 min (range: 61–105 min), and the mean intraoperative blood loss was 36.33 ± 7.41 mL (range: 20–50 mL). The mean hospitalization time was 5.13 ± 1.02 days (range3–7 days) (Table 2).

**Table 2:** Patient characteristics and surgical outcomes.

NO	Age (years)	Sex	Segment	F-up (months)	Approach	Operation time (min)	Blood loss (mL)	Hospitalization time (days)	Complication
1	47	M	T9/10	13	Single	65	30	6	None
2	50	F	T9/10	23	Single	76	40	5	None
3	57	F	T8/9	19	Single	70	30	3	None
4	62	F	T11/12	17	Single	74	40	5	None
5	68	F	T4/5	20	Double	101	45	7	Dura rupture
6	46	M	T11/12	25	Double	87	40	6	None
7	66	M	T10/11	22	Single	86	35	4	None
8	43	M	T8/9	18	Single	99	40	5	None
9	49	M	T7/8	26	Single	90	35	4	None
10	63	F	T10/11	22	Single	86	30	6	Dura rupture
11	51	F	T10/11	17	Single	78	30	5	None
12	60	F	T7/8	27	Single	96	35	6	None
13	70	M	T5/6	19	Single	98	45	5	None
14	55	F	T6/7	15	Double	105	50	6	None
15	64	F	T11/12	21	Single	61	20	4	None

The neurological symptoms of all patients improved after the operation, and their pain was significantly relieved. There were no cases of cerebrospinal fluid leakage, wound infection, or symptom progression. The mean follow-up time was 20.27 ± 3.87 months (range: 13–27 months). The mean JOA scores of patients improved from 5.53 ± 1.20 before the operation to 6.87 ± 1.31 after the operation and to 8.6 ± 1.25 at the last follow-up, with an average JOA improvement rate of 56.5 ± 18.00% and a significant difference between the preoperative and postoperative scores (*Z* = −3.437, *p* = 0.001). The JOA score improvement rate classification at the last follow-up was excellent in 3 cases, good in 8 cases, effective in 3 cases, and no change in 1 case, for an effective rate of 93.33%. The VAS score significantly improved from 6.67 ± 1.01 preoperatively to 3.47 ± 0.88 postoperatively and 1.73 ± 0.67 at the last follow-up, with an improvement rate of 74.38 ± 7.71% (*Z* = −3.497, *p* = 0.000). Additionally, the ODI significantly improved from 72.07 ± 6.08 preoperatively to 45.93 ± 5.01 postoperatively and 12.53 ± 2.33 at the last follow-up, with an improvement rate of 82.67 ± 2.38% (*t* = 46.398, *p* = 0.000) (Table 3).

**Table 3:** Preoperative and postoperative JOA, VAS and ODI scores.

NO	JOA			RR (%)	VAS			Improvement rate (%)	ODI			Improvement rate (%)
	Preop	Postop	Last		Preop	Postop	Last		Preop	Post	Last	
1	4	4	5	14.29	8	5	3	62.50	84	56	18	78.57
2	6	7	8	40.00	7	4	2	71.43	80	47	13	83.75
3	6	8	10	80.00	7	3	2	71.43	78	50	15	80.77
4	8	8	9	33.33	4	3	1	75.00	60	42	11	81.67
5	7	8	10	75.00	5	2	1	80.00	65	38	10	84.62
6	6	7	9	60.00	7	3	2	71.43	70	48	12	82.86
7	6	8	9	60.00	7	4	2	71.43	72	50	14	80.56
8	6	8	9	60.00	6	4	1	83.33	69	48	10	85.51
9	6	7	8	40.00	7	3	1	85.71	74	40	9	87.84
10	5	7	8	50.00	6	4	2	66.67	70	47	12	82.86
11	5	6	9	66.67	7	3	1	85.71	66	41	13	80.30
12	6	8	10	80.00	7	4	2	71.43	72	46	12	83.33
13	3	4	7	50.00	8	5	3	62.50	78	53	16	79.49
14	4	6	9	71.43	7	2	1	85.71	68	40	11	83.82
15	5	7	9	66.67	7	3	2	71.43	75	43	12	84.00
*p*-value		0.001	0.001			0.001	0.000			0.000	0.000	

The ossified posterior longitudinal ligament was completely removed in 13 patients, the dura was ruptured during the operation in 2 patients (patients 5 and 10), and neck discomfort occurred in 1 patient (patient 5), which was considered to be caused by high fluid pressure. Spinal cord hypertension was relieved by massage and by lowering the height of the irrigation fluid. The neurological symptoms of all patients improved after the operation, and their pain was significantly relieved. There were no cases of cerebrospinal fluid leakage, wound infection, or symptom progression.

## Discussion

The incidence of OPLL of the thoracic spine is low, at approximately 0%−1.9% (16), and it mainly occurs in the midthoracic region, which is poorly vascularized. Severe irreversible damage to the spinal cord can occur. Surgical removal of the ossified posterior longitudinal ligament and decompression of the ventral side of the spinal cord to restore spinal cord function are the main goals of its treatment (17, 18). The indications for spinal endoscopic technology are continuously expanding, and with this technology, diseased tissue can be excised accurately under direct endoscopic vision with relatively little damage to normal structures and satisfactory clinical efficacy. Many scholars have begun to try endoscopic-assisted retropleural approaches and transforaminal approaches for ventral spinal cord decompression in the treatment of TSS (19). In 2018, Ruetten et al. (20) first reported the application of an endoscopic-assisted retropleural approach in the treatment of TSS with ventral decompression of the thoracic spinal canal in adult cadavers. The results from the cadaveric study demonstrate that the transthoracic retropleural approach can provide easy access to the lesion site and allow adequate decompression of the prethoracic epidural space. This approach was applied in nine patients to decompress the ventral side of the spinal cord; while one of the eight patients who completed follow-up had a dural tear and two patients showed neurological deterioration at different times, the condition of all patients improved. Gao (21) and others described endoscopic-assisted transforaminal decompression of the ventral spinal cord, with improvements in the symptoms of all patients after surgery. The concept of a “safety triangle” has also been proposed; operating within the “safety triangle” can further improve the efficacy and safety of surgery. Adequate decompression of the ventral side of the spinal cord and reduced stimulation of the spinal cord are keys to ensuring surgical efficacy and safety. In this study, the clinical symptoms of the 15 patients were improved after surgery, and the imaging results showed adequate decompression of the spinal cord. The final JOA score of the patients was significantly improved compared with the preoperative score, and the effective rate was 93.33%. The final VAS score and ODI were significantly lower than those before surgery, and these results are similar to those of the other two surgical methods. However, the operative time in this study was 84.80 ± 13.23 min (range: 61–105 min), the mean intraoperative blood loss was 36.33 ± 7.41 mL (range: 20–50 mL), and the mean hospital stay was 5.13 ± 1.02 days. Compared with the traditional open surgery results reported by Masahiko et al. (22), all variables showed great improvement with this method. The follow-up results showed that the clinical efficacy of the transfacet joint approach was the same as that of the retropleural and transforaminal approaches and that satisfactory outcomes could be achieved. Therefore, the transfacet joint approach is safe and effective and can be used in the treatment of TSS caused by ventral spinal cord compression.

The classic surgical method for decompression of the ventral spinal cord in cases of compression caused by OPLL is the “box resection” method, in which anterior decompression is achieved without stimulating the spinal cord. However, anatomically, the thoracic cavity is immediately adjacent to the spinal canal, and the thoracic intervertebral foramen is largely occupied by the intercostal nerves. Thus, the conventional surgical approach for ventral spinal cord decompression may cause complications, such as pneumothorax or intercostal nerve stimulation or even injury, potentially affecting the surgical operation and even the therapeutic efficacy (23). The thoracic facet joint is located between the thoracic cavity and the spinal canal. During the operation, the endoscopic channel was placed on the dorsal side of the inferior articular facet, and then the upper and lower facet joints were trephined to access the ventral side of the spinal cord. There is a certain safe distance from the thoracic cavity that was maintained throughout the operation, and the intercostal nerve was basically not touched. Therefore, the transfacet joint approach can largely avoid the occurrence of such complications as those mentioned above. Zhao et al. (24) performed ventral spinal cord decompression in 14 patients through the transfacet joint approach. The only intraoperative complication was a dural tear, and no other complications, such as chest and nerve root injuries, occurred, which is consistent with the findings of this study. During decompression through the transfacet joint approach, part of the lamina on the dorsal side of the spinal cord can be removed first to achieve indirect decompression and further ensure the safety of ventral decompression operations. With the help of the 30° field of view of the spinal endoscope, approximately 180° of decompression can be achieved on the ventral side of the spinal cord; this scope of decompression is comparable to that of the transforaminal approach.

Intraoperative dural rupture has long been a common complication of spinal surgery. Cho et al. (25) reported that the incidence of cerebrospinal fluid leakage due to dural rupture is as high as 37.7%. The incidence of dural rupture is related to dural ossification. Yu et al. (26) reported that the incidence of cerebrospinal fluid leakage was 63.6% and 3.5% in those with and without dural ossification, respectively, and that gelatin foam can be applied for dural rupture repair during the operation. The dural rupture rate in the present study was 13.3%, which is similar to that reported in the literature and may be due to the small sample size of our study. It is also possible that our use of a drill to fully remove the ossified tissue by grinding contributed to this result. Mazur (27) and others believe that although direct dural repair is the preferred treatment for cerebrospinal fluid leakage, conservative measures are also feasible when the dura cannot be directly repaired. Due to their small size, dural ruptures that occur during endoscopic surgery are difficult to repair. Therefore, in patients with a dural rupture, we applied a dural repair material or gelatin foam and did not place a drainage tube after surgery; no symptoms of low intracranial pressure (such as headache and vomiting) were observed.

Endoscopic-assisted ventral decompression of the thoracic spinal cord has high risks and is difficult, and reducing surgical complications and improving surgical efficacy remain continuous pursuits. Therefore, many new technologies continue to be applied in surgery. In 2019, Zhang et al. (28) applied a new flexible burr system under endoscopy to treat 11 patients with calcified thoracic disc herniation, and no postoperative complications were observed. Alcachupas (29) described the use of a 3D intraoperative imaging system in surgery for patients with thoracic disc herniation. New technologies, such as intraoperative ultrasound technology, have also been used (30). The use of visualization technologies and other new technologies in the treatment of TSS overcomes the limitations of thoracic spinal surgery, improves surgical safety while improving surgical efficacy, and facilitates ventral thoracic spinal cord decompression by decreasing the difficulty and complexity of the involved surgical procedures.

## Conclusion

The classic surgical method for ventral spinal cord decompression in cases of compression caused by OPLL is the “box resection” method, in which anterior decompression is achieved without stimulating the spinal cord. The new minimally invasive procedure for resecting the OPLL described herein yielded excellent postoperative outcomes with few complications, demonstrating its safety and effectiveness. However, the sample size of this study was small, the follow-up time was short, and the long-term efficacy of the method requires verification by long-term follow-up observation.

## Data Availability

The raw data supporting the conclusions of this article will be made available by the authors, without undue reservation.
